# The Implementation of a Perioperative Registry in a Resource-Limited Setting: A Feasibility, Fidelity, and Acceptance Study

**DOI:** 10.18103/mra.v13i6.6574

**Published:** 2025-06-30

**Authors:** Joshua D Gazzetta, Poster P Mutambo, Mutimba B Mpabalwani, Mwamba JC Mulenga, Cyrus Phiri, Kelvin Shaba, Emmanuel M Makasa

**Affiliations:** 1 Department of Surgery, University of Virginia School of Medicine, Charlottesville, VA; 2 Department of Surgery, University Teaching Hospital, Ridgeway, Lusaka, Zambia; 3 Centre for Surgical Healthcare Research (CSHR)

## Abstract

**Background:**

In low and middle-income countries, nine out of every ten persons is unable to access safe and timely surgery. The limited perioperative data in resource-limited settings compromises surgical and research capacity growth. By increasing data availability, surgical disparities may be addressed through research efforts and quality initiatives. This project aimed to implement and evaluate a perioperative registry in a tertiary care hospital in a low-income country.

**Methods:**

A prospective emergency laparotomy perioperative registry was implemented in Zambia’s largest teaching and referral hospital. Over the first 6-months of implementation, 162 patients were included. Data was collected postoperatively, before discharge, and at 30 days. The registries feasibility was assessed by evaluating patient accrual, retention, and 30-day completion rates. The registries fidelity was measured by evaluating data missingness. A participant acceptance survey was retrospectively collected and analyzed for the first 25 consecutively enrolled patients.

**Results:**

The capture rate of the registry could not be calculated due to a destroyed theatre logbook. The participant accrual and retention rates were 99.4% and 95.1%, respectively. The participant completion rate at 30-days was 75.6%. The overall incidence of missing information in the registry was 3.5%. More than 75% of participant responses to the acceptance survey were positive in each category regarding the ethical conduct of research and the storing of personal data.

**Conclusion:**

The value of this study is the reporting and evaluation of a successful perioperative registry implementation with minimal external funding. This framework is being used to develop new data registries and may provide a roadmap for other hospitals with resource constraints.

## Introduction

The global health burden from surgical disease is nearly 30%. Surgical disparities are greatest in low and middle-income countries (LMICs), where nine out of ten people cannot access safe and timely surgery.^1–5^ Further, perioperative mortality rates are three times higher in LMICs when compared to high-income countries (HICs).^6^ Over the last decade, disparities in surgical healthcare have become better recognized and there has been a shift toward prioritizing surgical capacity building in resource-limited settings.^7^ Even with increased global awareness of unmet surgical needs worldwide, the translation of advocacy efforts into meaningful change has been slow and the investments limited.^8^ There is an urgent need to identify cost-effective strategies to define gaps in surgical care in low-resource settings.

The importance of perioperative data to build surgical capacity is highlighted at the global, continental, and national levels by the Lancet Commission on Global Surgery (LCoGS), The African Peri-operative Research Group (APORG), and The Republic of Zambia’s National Surgical Obstetric and Anaesthesia Plan (NSOAP), respectively.^2,9^ While HICs have demonstrated improved perioperative outcomes through quality initiatives, utilizing the systematic collection of surgical outcomes data (SOD), perioperative data collection in LMICs remain sparse.^10^ Limited access to perioperative data compromises the ability to track outcomes, develop risk-prediction models, practice evidenced-based care, and build research capacity. Additionally, continuous data is needed to report on the core surgical indicators developed by the global community to inform national policy and measure surgical capacity strength.^2,11,12^ With a need for high-quality research to improve surgical disparities in LMICs, a focus needs to be placed on local research capacity building, collaboration, and the use of technology.^13^

At the University Teaching Hospitals (UTH) in Lusaka, Zambia, perioperative data are recorded on paper charts and returned to the patient on discharge. This process leaves a gap in available data for research and patient improvement initiatives. A practical and ethical perioperative registry, suitable to the local context, is needed to drive improvement in research and to benchmark strengths and weaknesses of surgical care at UTH. Patients’ willingness to engage in research is also needed to understand the acceptability of data registry initiatives and research within the local population.

This project aims to implement and evaluate a perioperative registry for all patients undergoing emergency abdominal surgery (emergency laparotomy) to provide evidence for local researchers, inform providers and the health system of disparities in emergency laparotomy care, and provide a framework for surgical quality improvement. This project also aims to establish an outline that may be used for other surgical specialties at UTH and other LMIC hospitals without electronic medical records. The registry creation is part of an ongoing prospective study assessing the epidemiology and 30-day outcomes of emergency laparotomy patients at UTH (ELOP-study). We report on the design, implementation, and participant acceptance of a perioperative data registry for the largest teaching and referral hospital in Zambia.

## Methods

This project was approved by the National Health Research Authority (NHRA) and the University of Zambia (UNZA) Biomedical Research Ethics Committee (REC). Additionally, administrative approvals were given from the Lusaka Provincial Health Office (PHO) and the Senior Medical Superintendent’s office on behalf of the Ministry of Health (MOH). Informed consent was provided by the research participants.

This study was designed to implement a prospective perioperative registry and evaluate the feasibility, fidelity, and acceptance of implementation. The registry was created in Research Electronic Data Capture (REDCap) by the research team composed of members from the Center for Surgical Healthcare Research (CSHR), which is housed in UTH’s Department of Surgery. The research group is led by a Professor of Surgery with global surgery experience as a consultant and diplomat for the Southern African Development Community member-states, the World Health Organization and the United Nations and includes a research fellow with REDCap experience and a junior resident medical officer (JRMO) from the University of Zambia (UNZA). The JRMO was chosen for data collection and was given a stipend to assist with all aspects of the project using external funding.

### CHOICE OF mHEALTH PLATFORM AND DATA COLLECTION PROCESSES

The database platform was chosen using the guiding principles of (1) patient confidentiality (2) ease of use, (3) costs, and (4) functionality. REDCap is a web-based application created at Vanderbilt University in 2004 for research and database management. REDCap meets all Health Insurance Portability and Accountability Act standards.^14^ Other available mHealth platforms, including Smartcare and DHIS2, were considered but are not currently available to collect comprehensive perioperative data at UTH.

Starting in April of 2024, patients were consented and interviewed following an emergency laparotomy. During the first 6-months of participant enrollment, 162 patients were identified for inclusion. All three team leads piloted data collection together for the first week. REDCap instruments and variables were then adjusted and the database variable sets were finalized. The JRMO and the fellow with REDCap experience collected data together for an additional month until the primary data collector was comfortable collecting data on his own. Study participants were identified by the paper theatre logbook, recruited consecutively, and followed for 30 days. Postoperative, pre-discharge, and postoperative day 30 interviews were conducted. If a participant was discharged before 30 days, a phone interview was conducted. Three attempts were made to contact each patient by phone for the 30-day interview. Participants who underwent non-emergency abdominal surgery, were less than 18 years of age, pregnant, or incarcerated were excluded. The team leads hold weekly meetings and perform REDCap entry audits to assess the quality of the registry. Additionally, the research team created a Likert survey to evaluate participant acceptability of the ethical implementation of the registry. This survey was administered to the first 25 eligible participants retrospectively.

### VARIABLES

In concert with the prospective ELOP-study, the variables collected were chosen to assess the epidemiology and 30-day outcomes of emergency laparotomy patients presenting to UTH. Insights from local champions within the department of surgery and CSHR were provided to determine what necessary variables could be accurately found from the medical chart and through patient interviews. Postoperative variables collected included name, phone number, date of surgery, age, sex, home province, residential area, insurance status, education level, employment, income, comorbid conditions, smoking history, alcohol use, HIV status, history of abdominal surgery, referral status, referral hospital, time spent at the referral hospital, time from symptom onset to the patient’s decision to seek treatment, time from the patient’s decision to seek treatment to hospital presentation, time from UTH presentation to the operating theatre, transport types, transportation times, Qsofa score on arrival, laboratory analyses, surgery indication, surgery performed, surgeon level of training, surgery time of day, surgery duration, and tumor status. Prehospital discharge variables included mortality, length of stay (LOS), relaparotomy, ICU admissions, blood transfusion needs, and postoperative complications. Thirty-day follow-up variables included readmissions and postoperative complications, including surgical site infections.

### ANALYSIS

The capture rate was to be assessed by calculating the percentage of patients captured in REDCap compared to the operating theatre log. However, a large portion of the theatre logbook was destroyed during the study period preventing an accurate calculation. To evaluate the fidelity of the dataset, data missingness was calculated by analyzing the percent missing data for each variable collected at the postoperative, pre-discharge, and 30-day interview intervals. Participants with in-hospital mortality were excluded from the 30-day interview data missingness calculations.

To determine the feasibility of an ongoing perioperative registry at UTH, patient accrual rates (participants willingness to consent to the postoperative interview), retention rates (participant willingness and availability to provide data at the pre-discharge interview), and completion rates (participant willingness and availability to provide information at the 30-day interview) were calculated as percentages. Participants with in-hospital mortality were also excluded from the 30-day interview feasibility calculations. The Likert survey results on participant acceptability are reported in percentages.

## Results

**Figure 1:**
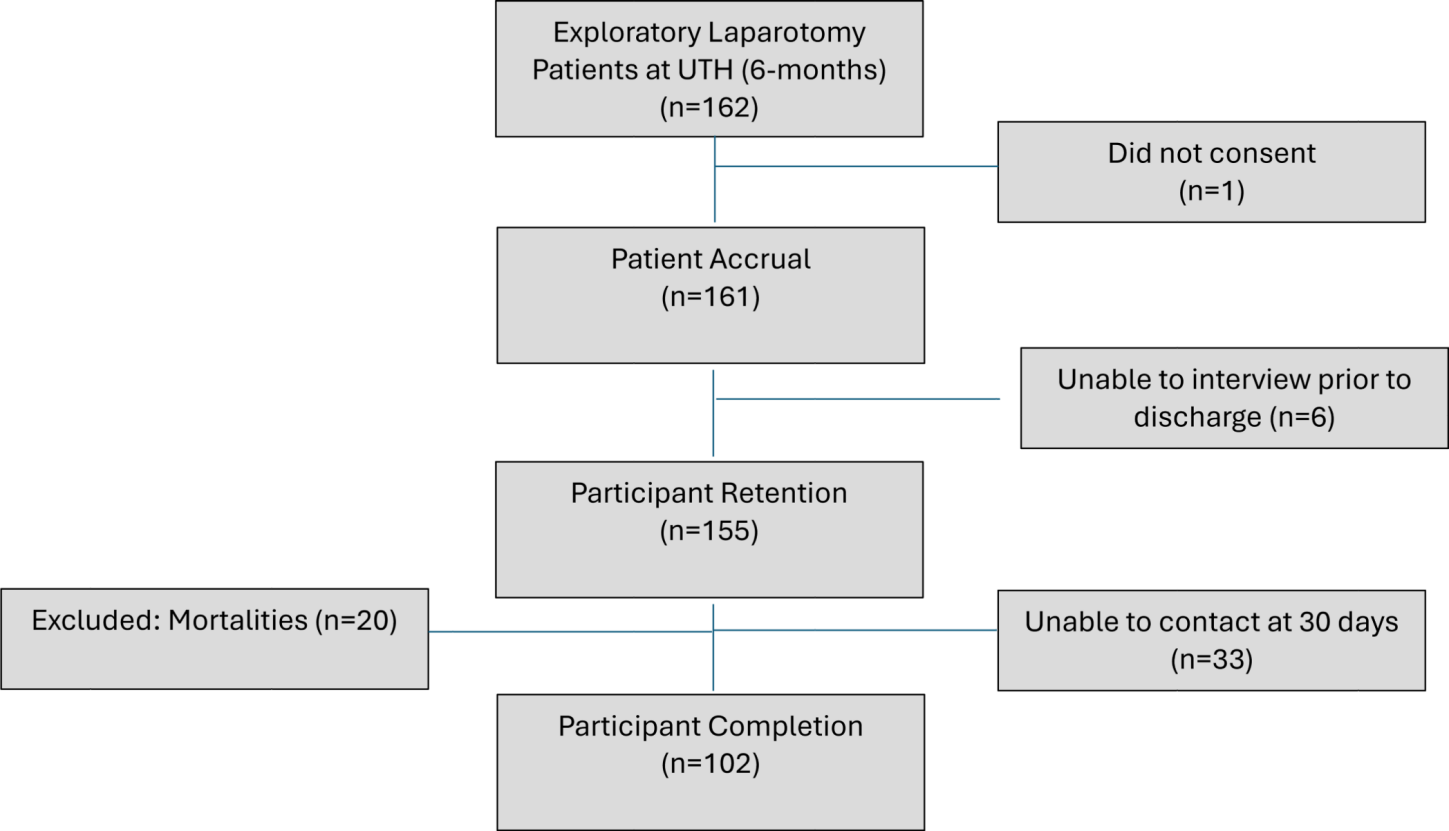
Participant accrual, retention and completion (feasibility)

The capture rate was not calculated due to the theatre logbook being destroyed. An accrual rate of 99.4% was found with 161 of 162 participants willing to consent to the postoperative interview. A 95.1% retention rate was found with 6 participants discharged before the prehospital interview and could not be located for further review. Twenty inpatient mortalities were excluded. Of the 135 remaining participants, 102 completed the 30day interview for a 75.6% completion rate. Overall, 102 of 142 (71.8%) included participants completed all three interviews (Figure 1).

The registry’s fidelity assessment, as seen in Table 1, reveals the highest data missingness was found in the 30-day interview, with 20.1% and 22.8% missingness of variables. Intraoperative tumor findings (9.3% missing), preoperative creatinine level (7.4% missing) and surgery time of day (7.4% missing) had the next highest missingness. The overall incidence of missing data for consenting participants was 3.5%.

The acceptability survey results in Table 2 show the reaction of participants to having their data placed into a registry. While 16% of patients were “not confident at all” or “somewhat confident” their personal information would be kept confidential, 75% or more provided positive responses in all categories answering questions with “clear or very clear,” “often or always,” “confident or very confident,” and “well or very well.”

When asked if the patient had any additional comments, two common themes emerged. The first was “thank you,” and the second was “I feel important.” One negative comment of “not satisfied” was recorded.

## Discussion

A prospective perioperative registry was successfully created for emergency laparotomy patients in a tertiary teaching hospital in Lusaka, Zambia. While the capture rate could not be calculated due to a destroyed theatre log book, the incidence of missing data was 3.5%, revealing a high-fidelity registry. The destroyed theatre logbook provides additional evidence for the need for sustainable electronic medical record keeping. Additionally, the high feasibility, measured by patient accrual, retention, and completion rates, highlights how this method of registry implementation may be viable long-term in the local context. The participant acceptability survey results uncover an overall satisfaction with the conduct of a registry implementation and shed light on areas for improved research participant education. In the future, more patient and public involvement (PPI) in research design and intervention will help engage the local community. Using participatory research methods may promote more patient engagement and equity in the research process.^15^ Additionally, we plan on spending more time educating participants about the confidentiality of their healthcare information during the consenting process as the database continues. To our knowledge, this is the first study to evaluate the implementation of a perioperative data registry in Zambia and the first study to evaluate the ethical conduct of research and registry creation from the participants’ vantage point in Sub-Saharan Africa.

The perioperative registry creation answered several important questions, including that UTH has the human and technological resources to house a registry, participants are willing to engage in research and have personal data stored, and minimal external funding is needed to sustain ongoing data collection. While many LMICs do not track perioperative outcomes for 30-days, it is a recognized benchmark for surgical quality, and we found that it was feasible in the local surgical population using telephone interviews.^16^ A similar method for perioperative registry creation was demonstrated in Hawassa, Ethiopia using the REDCap platform. The authors also report a successful registry creation with minimal external funding.^17^ Few challenges were met implementing our registry with the primary data collector reporting REDCap was easy to use and minimal time obligations were needed for data entry while maintaining other duties in the hospital. Further, the REDCap rights are owned by the MOH and registry-building has local support from all parties invested in patient outcome improvement, including the community and clinical and administrative staff.

Despite the growing body of evidence supporting electronic medical registry creation in low-resource settings, they remain relatively uncommon.^18,19^ In Zambia, just 2% of hospitals use electronic systems as their sole source for patient record keeping.^20^ The inability to measure data continuously prevents clinicians and stakeholders from developing priorities for patient care improvement and creates a challenge for health system infrastructure growth.^21^ Some of the barriers to registry creation in LMICs include cost, technology infrastructure, complexity of data collection instruments, and challenges with data quality due to incomplete data. Additionally, the emotional impact of documenting adverse events among providers has been shown as a barrier to registry implementation.^19,22^ To overcome some of these obstacles, it will be important to maintain existing local workflows, retain access to low-cost technology such as REDCap, empower data collectors, keep data collection tools simple and organized, and minimize breaches of patient confidentiality.

Future challenges of data registries specific to UTH include sustainability after external funding is no longer available. To combat this, the CSHR has offered to provide data collectors as part of their research curriculum, allowing junior researchers to gain skills in technology, patient interviewing, and research project development. An additional challenge is missing data from either incomplete documentation or the lack of standardized reporting. For example, several variables chosen for this registry were largely unavailable, including surgery duration, due to incomplete documentation. This provides an opportunity for the surgery department to develop initiatives for uniform reporting and documentation standardization.

A longer-term goal following this study is to modify this registry into a more comprehensive perioperative database to include all surgical patients throughout the continuum of care. This will allow for better outcome reporting, research capacity, data for evidence-based perioperative care, and quality initiative development. In addition, data may be used to benchmark surgical priorities set by global and national stakeholders and inform the Republic of Zambia’s NSOAP revisions. With the creation of this registry, there has been an overall positive response within the surgery department and new registries are currently being created for burn patients, brain tumor patients, and road traffic accident victims. Two of the three new registries are being spearheaded by JRMOs interested in learning the process from start to finish.

The first limitation of this study is the missing capture rate. However, the destruction of the theatre logbook is an important finding and an additional reason to continue electronic medical record keeping. Another limitation stems from this study being performed in concert with the ELOP-study. The variable set was designed to evaluate only one cohort of surgery patients and it is uncertain if a more robust variable set would be collected as successfully. It is also unknown if this registry has high transference to other low-resource settings, as successful implementation has been found in countries with engaged participants and organized research teams.^18^ As UTH develops new registries, formal research curriculums and data quality checks need to be bolstered.

There is a vast difference in healthcare infrastructure, workforce capacity, resources, and service delivery in LMICs compared to HICs and quality initiatives are not always transferrable between the two. More access to data in low-resource areas provides opportunity to collect ideas for change, benchmark progress, and improve patient outcomes in the local context. While there is little data available for emergency surgery outcome research in LMICs, other fields in low-resource settings have been able to leverage data to improve quality of surgical care. For example, one study found that implementing the International Quality Improvement Collaborative, developed in Pakistan, reduced surgical site infections and sepsis rates among patients undergoing congenital heart surgery.^10^ Identifying ways to systematically collect data is possible and necessary to employ data-driven improvement in surgical care.

## Conclusion

This study provides data on the feasibility, fidelity, and acceptability of a perioperative database implementation for emergency laparotomy patients in a resource-limited setting. It may also provide a roadmap for registry creation for other hospitals with limited human, technology, and financial resources.

## Figures and Tables

**Table 1:** Data Missingness (fidelity)

Variables	Missingness n (%)
Postoperative Variables	
Name	0 (0%)
Phone number	4 (2.5%)
Date of surgery	0 (0%)
Age	0 (0%)
Sex	0 (0%)
Home province	1 (0.62%)
Residential Area	1 (0.62%)
Insurance status	5 (3.1%)
Education level	6 (3.7%)
Employment	5 (3.1%)
Income	5 (3.1%)
Comorbid conditions	4 (2.5%)
Smoking history	4 (2.5%)
Alcohol use	4 (2.5%)
HIV status	6 (3.7%)
History of abdominal surgery	11 (6.8%)
Referral status	2 (1.2%)
Referral hospital	1 (0.62%)
Time spent at the referral hospital	4 (2.5%)
Time from symptom onset to patient decision to seek treatment	4 (2.5%)
Time from decision to hospital presentation	3 (1.9%)
Time from UTH arrival to operating theatre	9 (5.6%)
Transportation type	3 (1.9%)
Transportation time	3 (1.9%)
Qsofa score on arrival	7 (4.3%)
Preoperative albumin level	8 (4.9%)
Preoperative hemoglobin level	5 (3.1%)
Preoperative creatinine level	12 (7.4%)
Preoperative platelet count	4 (2.5%)
Preoperative white blood count	4 (2.5%)
Surgery indication	0 (0%)
Surgery performed	0 (0%)
Surgeon level	6 (3.7%)
Surgery time	12 (7.4%)
Surgery duration	3 (1.9%)
Tumor status	15 (9.3%)
Pre-discharge variables	
Mortality	6 (3.7%)
LOS	6 (3.7%)
Relaparotomy	6 (3.7%)
ICU admission	9 (5.6%)
Blood transfusion	5 (3.1%)
Postoperative complications	6 (3.7%)
30-day follow-up variables	
Readmitted	33 (20.1%)
Postoperative complications	37 (22.8%)

**Table 2:** Participant Acceptability

Questions	Answers n (%)				
	Very unclear	Unclear	Neutral	Clear	Very clear
**Was the informed consent process clear and easy to understand?**	1 (4%)	2 (8%)	2 (8%)	9 (36%)	11 (44%)
**Were all the procedures of the study clearly explained to you?**	0 (0%)	0 (0%)	1 (4%)	11 (44%)	13 (52%)
	Never	Rarely	Sometimes	Often	Always
**Did you feel that your participation in the study was completely voluntary?**	0 (0%)	1 (4%)	0 (0%)	7 (28%)	17 (68%)
**Did you feel respected and treated fairly during your participation in the study?**	0 (0%)	1 (4%)	0 (0%)	4 (16%)	20 (80%)
**Were you given the opportunity to provide feedback during the study?**	2 (8%)	0 (0%)	2 (8%)	9 (36%)	12 (48%)
**Were your concerns and questions adequately addressed by the study team?**	0 (0%)	0 (0%)	4 (16%)	6 (24%)	15 (60%)
	Not confident at all	Somewhat confident	Neutral	Confident	Very confident
**Were you confident that your personal information would be kept confidential?**	2 (8%)	2 (8%)	2 (8%)	9 (36%)	10 (40%)
	Not at all	A little	Neutral	Well	Very well
**Did you feel that the data collected during the study was securely handled?**	0 (0%)	3 (12%)	1 (4%)	11 (44%)	10 (40%)
